# Supplementary material for: Assembly defects of human tRNA splicing endonuclease contribute to impaired pre-tRNA processing in pontocerebellar hypoplasia
Source: Nat Commun. 2021 Sep 28;12:5610. doi: 10.1038/s41467-021-25870-3 (PMC8479045; doi:10.1038/s41467-021-25870-3)
Supplement: Supplementary file 1 — Supplementary Information [file 41467_2021_25870_MOESM1_ESM.pdf]

Supplementary Information

Assembly defects of human tRNA splicing endonuclease contribute to impaired pre-tRNA processing in pontocerebellar hypoplasia

Samoil Sekulovski[1†], Pascal Devant[1,2,3†], Silvia Panizza[4†], Tasos Gogakos[5], Anda Pitiriciu[1], Katharina Heitmeier[1], Ewan Phillip Ramsay[6], Marie Barth[7], Carla Schmidt[7], Thomas Tuschl[5], Frank Baas[8], Stefan Weitzer[4], Javier Martinez[4*] & Simon Trowitzsch[1*]

[1] Institute of Biochemistry, Biocenter, Goethe University Frankfurt, Max-von-Laue Strasse 9, 60438 Frankfurt/Main, Germany.

[2] Ph.D. Program in Virology, Harvard Medical School, Boston, MA 02115, USA.

[3] Harvard Medical School and Division of Gastroenterology, Boston Children's Hospital, Boston, 300 Longwood Avenue, MA 02115, USA.

[4] Max Perutz Labs, Medical University of Vienna, Vienna Biocenter (VBC), Dr. Bohr-Gasse 9/2, 1030 Vienna, Austria.

[5] Laboratory for RNA Molecular Biology, The Rockefeller University, 1230 York Avenue, New York, NY 10065, USA.

[6] The Institute of Cancer Research, 237 Fulham Road, London, SW3 6JB, United Kingdom

[7] Interdisciplinary research center HALOmem, Charles Tanford Protein Center, Institute of Biochemistry and Biotechnology, Martin Luther University Halle-Wittenberg, Kurt-Mothes-Strasse 3a, 06120 Halle, Germany.

[8] Department of Clinical Genetics, Leiden University, Albinusdreef 2, 2333 ZA Leiden, Netherlands.

[†] These authors contributed equally: Samoil Sekulovski, Pascal Devant, Silvia Panizza.

* Corresponding authors: J.M. (e-mail: javier.martinez@meduniwien.ac.at),
S.T. (e-mail: trowitzsch@biochem.uni-frankfurt.de)

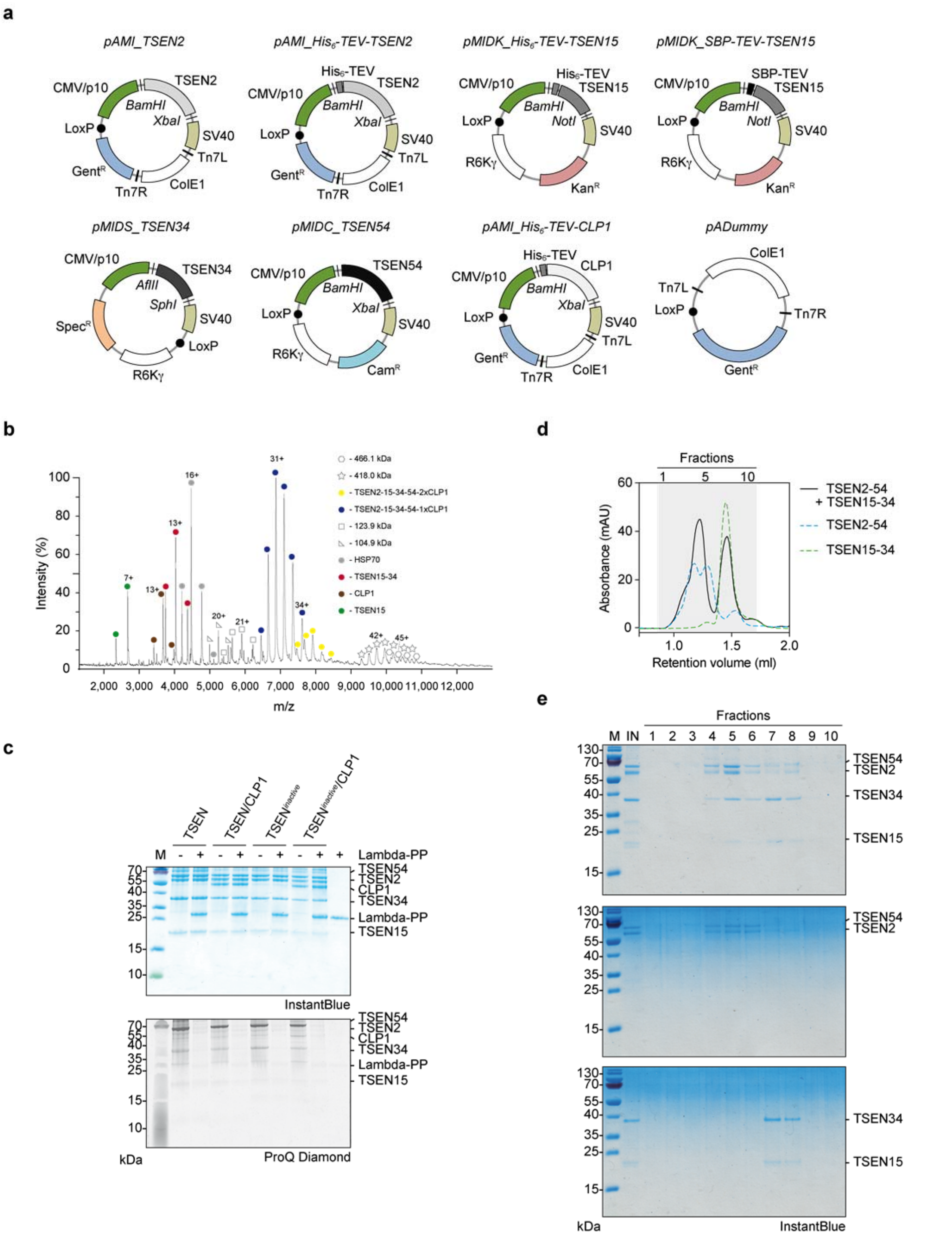

Supplementary Figure 1. **Biochemical characterization of recombinant TSEN and TSEN/CLP1 complexes. a** Maps of modified MultiBac vectors encoding TSEN/CLP1 complex components. For expression in mammalian and insect cells, the acceptor vector pAMI and donor vectors pMIDC, pMIDK, and pMIDS carry the CMV/p10 dual promoter. Transcription is terminated by the SV40 poly-A late signal (SV40). The transposase elements Tn7L and Tn7R, the LoxP element (black dot) for Cre-

mediated recombination, the origins of replication ColE1 and R6Kγ, and the resistance markers for gentamicin ($Gent^R$), chloramphenicol (Cam^R), kanamycin, (Kan^R), and spectinomycin ($Spec^R$) are shown. Restriction sites, hexahistidine-tags (His_6), the Streptavidin-binding peptide-tag (SBP), and the TEV cleavage site (TEV) are indicated. **b** Native mass spectrum of pentameric TSEN/CLP1 complex from an aqueous ammonium acetate solution. Charge states of the predominant TSEN/CLP1 assembly (blue circles), a minor populated TSEN complex with two CLP1 subunits (yellow circles), monomeric CLP1 (brown circles), HSP70 (grey circles), the TSEN15–34 heterodimer (red circles) and TSEN15 (green circles) are indicated. Unidentified protein assemblies are denominated by their molecular weights. **c** Analysis of phosphorylation states of TSEN components by the phospho-specific ProQ Diamond gel stain. The strong band at 59 kDa in the ProQ Diamond stain corresponds to TSEN54. Lambda-PP, Lambda protein phosphatase. Gels are representative of three independent experiments. **d** Assembly assay with TSEN2–54 and TSEN15–34 heterodimers via size exclusion chromatography (SEC). Absorbance profiles (280 nm) of reconstituted TSEN complex (black line), and the heterodimers TSEN2–54 (blue dashed line) and TSEN15–34 (green dashed line) are shown. **e** SDS-PAGE of SEC fractions (grey area as indicated in **d**) with subsequent InstantBlue staining. Gels are representative of two independent experiments. Source data for **c** and **e** are provided as Source Data file.

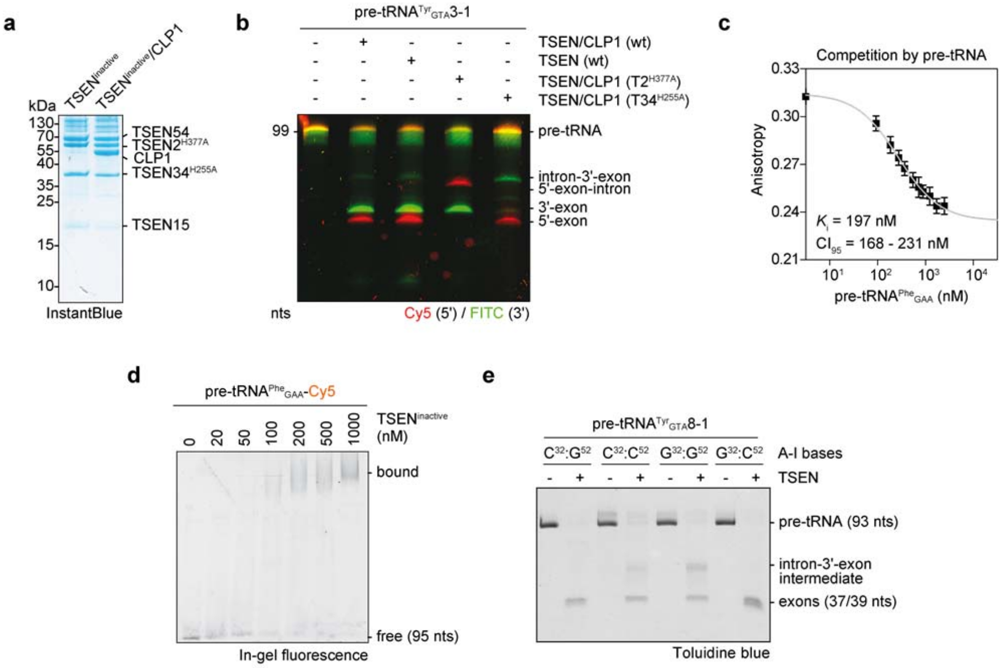

Supplementary Figure 2. **Active involvement of the A–I base pair in coordinating pre-tRNA cleavage.** **a** SDS-PAGE of purified, recombinant inactive TSEN and inactive TSEN/CLP1 complexes ($TSEN2^{H377A}$ and $TSEN34^{H255A}$ double mutant). Protein size markers and protein identities are indicated. **b** Two-colored pre-tRNA cleavage assay with TSEN-STREP and TSEN/CLP1-FLAG wt complexes and complexes carrying the $TSEN2^{H377A}$ ($T2^{H377A}$) or $TSEN34^{H255A}$ ($T34^{H255A}$) substitution. RNA cleavage products were separated on a denaturing urea-PAGE and visualized by fluorescence of cyanine5 (Cy5) and Fluorescin (FITC). **c** Thermodynamic competition parameters deduced from fluorescence anisotropy experiments. Inactive, tetrameric TSEN bound to fluorescently labeled pre-$tRNA^{Phe}_{GAA}$ was titrated with unlabeled pre-tRNA. Data are represented as mean values ±SD. **d** Electrophoretic mobility shift assay with fluorescently labeled pre-$tRNA^{Phe}_{GAA}$ and inactive, tetrameric TSEN ($TSEN^{inactive}$). Free and bound fractions of pre-tRNA were analyzed via 4% TBE native PAGE with subsequent in-gel fluorescence measurement. **e** Impact of A-I base pair mutations in pre-$tRNA^{Tyr}_{GTA}$8-1 on endonucleolytic activity by tetrameric TSEN revealed by a pre-tRNA cleavage assay. CI_{95} – 95% confidence interval, C^{32}:G^{52} – canonical A-I base pair, C^{32}:C^{52} and G^{32}:G^{52} – disrupted A-I base pair, G^{32}:C^{52} – inverted A-I base pair. Panels are representatives of three independent experiments. Source data for **a**, **b**, **d**, and **e** are provided as Source Data file.

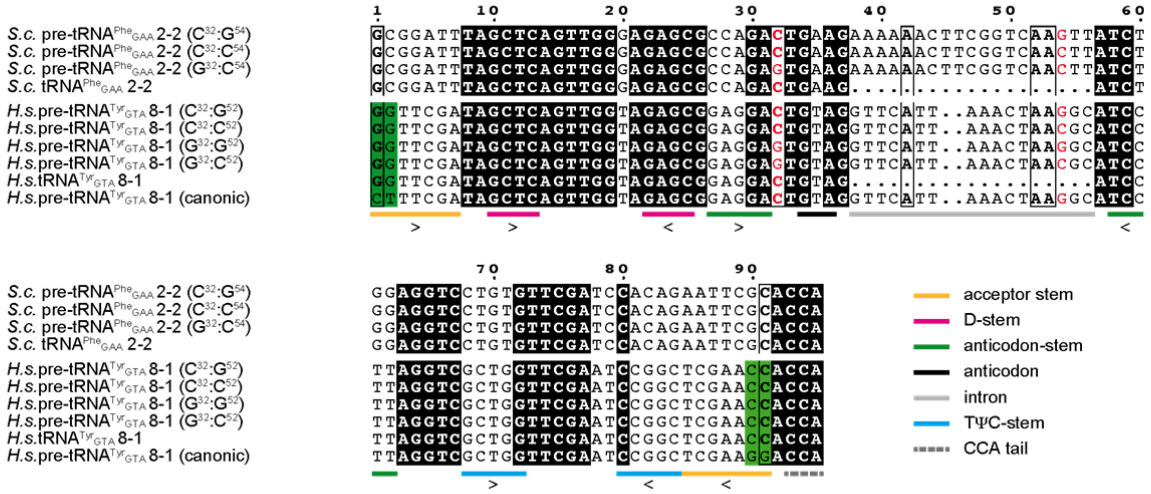

Supplementary Figure 3. **Sequence comparison of pre-tRNA and tRNA molecules.** Sequence alignments were performed using Clustal Omega, edited in Jalview and colored by conservation using ESPript 3.0. A–I base pair residues are colored in red. Predicted stem structures, anticodon, intron and CCA tail are indicated by colored bars. Ribonucleotides modified for efficient *in vitro* transcription are boxed in green and compared to the canonical sequence.

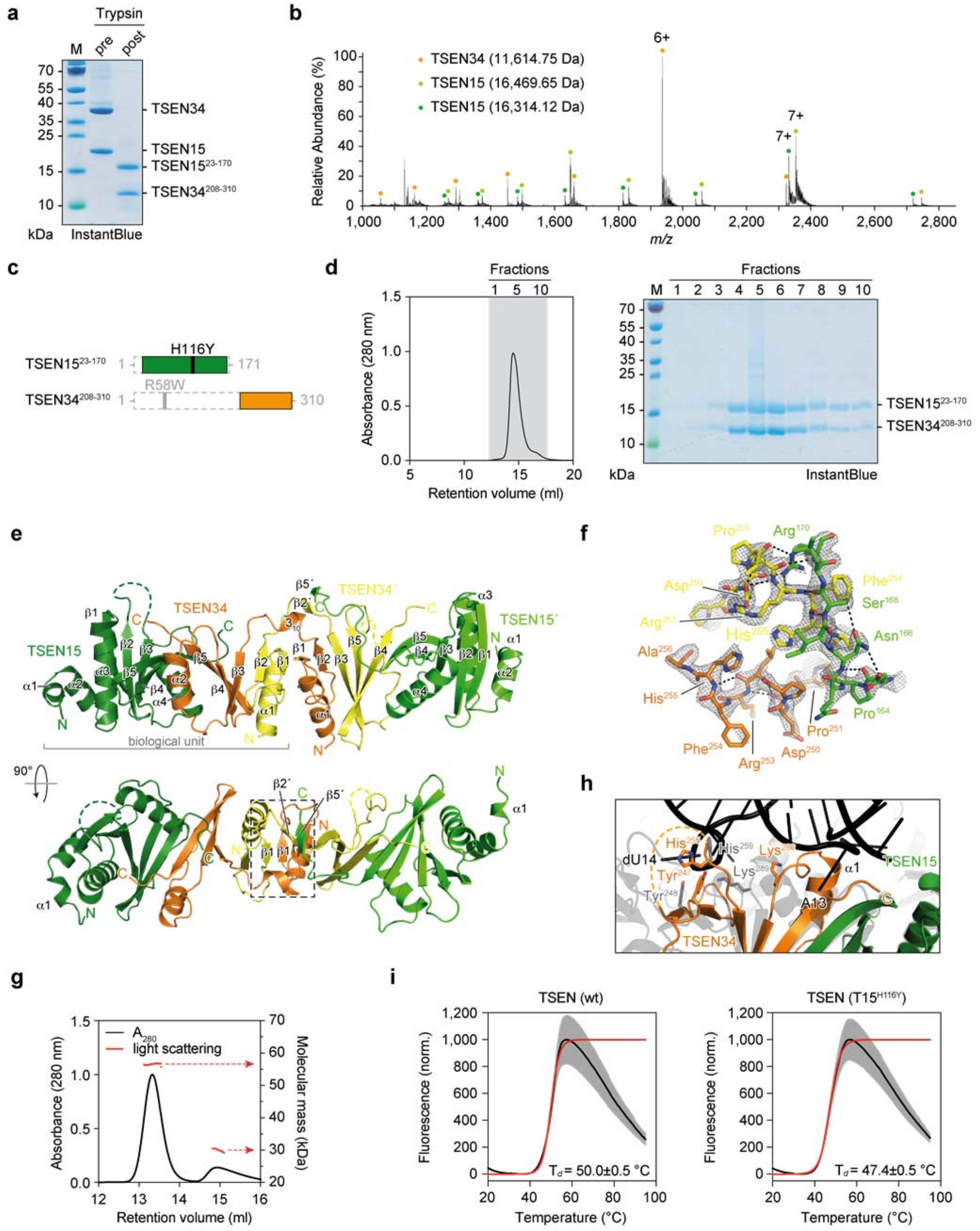

Supplementary Figure 4. **X-ray crystal structure of a TSEN15–34 heterodimer derived by limited proteolysis. a** Analysis of the limited tryptic digestion of the TSEN15–34 heterodimer by SDS-PAGE. **b** Denaturing mass spectrum of the proteolytically stable fragments of the TSEN15–34 heterodimer from an aqueous ammonium acetate solution. The mass spectrum shows the presence of a TSEN34 fragment (orange circles), and two TSEN15 fragments (dark and light green circles) differing in mass only by a C-terminal arginine as revealed by LC-MS/MS. **c** Bar diagrams of tryptic fragments of TSEN15 and TSEN34. Proteolyzed regions are indicated by dashed boxes. Positions of PCH

mutations are shown. **d** Purification of the re-cloned core of the TSEN15–34 heterodimer via SEC. The absorbance profile at 280 nm is shown. Fractions of the indicated retention range (grey area) were analyzed by SDS-PAGE. **e** Asymmetric unit of the TSEN15–34 crystal. The biological unit (bracket) and the domain-swap area (dashed box) are indicated. α-helices and β-sheets are numbered for each subunit. **f** Stick representation of amino acids of the domain-swap area with electron density ($2F_o$-F_c, 1.5σ). **g** Molecular mass determination by size exclusion chromatography multi-angle light scattering (SEC-MALS) of the TSEN15–34 sample used for crystallization. The data reveal a dominant population of a dimer-of-a-heterodimer (13.2 ml, 56.6 kDa) and a minor populated heterodimer (14.9 ml, 30.0 kDa). Light scattering is shown as red dots. Mass determination by SEC-MALS was confirmed by two independent experiments. **h** Superposition of the TSEN15–34 heterodimer and the pre-tRNA endonuclease from *Archaeoglobus fulgidus* at the interaction sites with the bulge-helix-bulge RNA (PDB ID 2GJW). Nucleotide positions of the RNA (black) and residues of the catalytic triads are shown. **i** Representative thermal denaturation curves (n=2) as shown in Fig. 3g of recombinant wt TSEN and mutant TSEN ($T15^{H116Y}$) complexes derived from DSF. Sigmoidal Boltzmann fits are shown as red lines (black lines represent mean values). Grey zones show SDs from technical triplicates. Denaturation temperature (T_d) is presented with error of fit. Gels shown in **a** and **d** are representative of three independent experiments. Source data for **a** and **d** are provided as Source Data file.

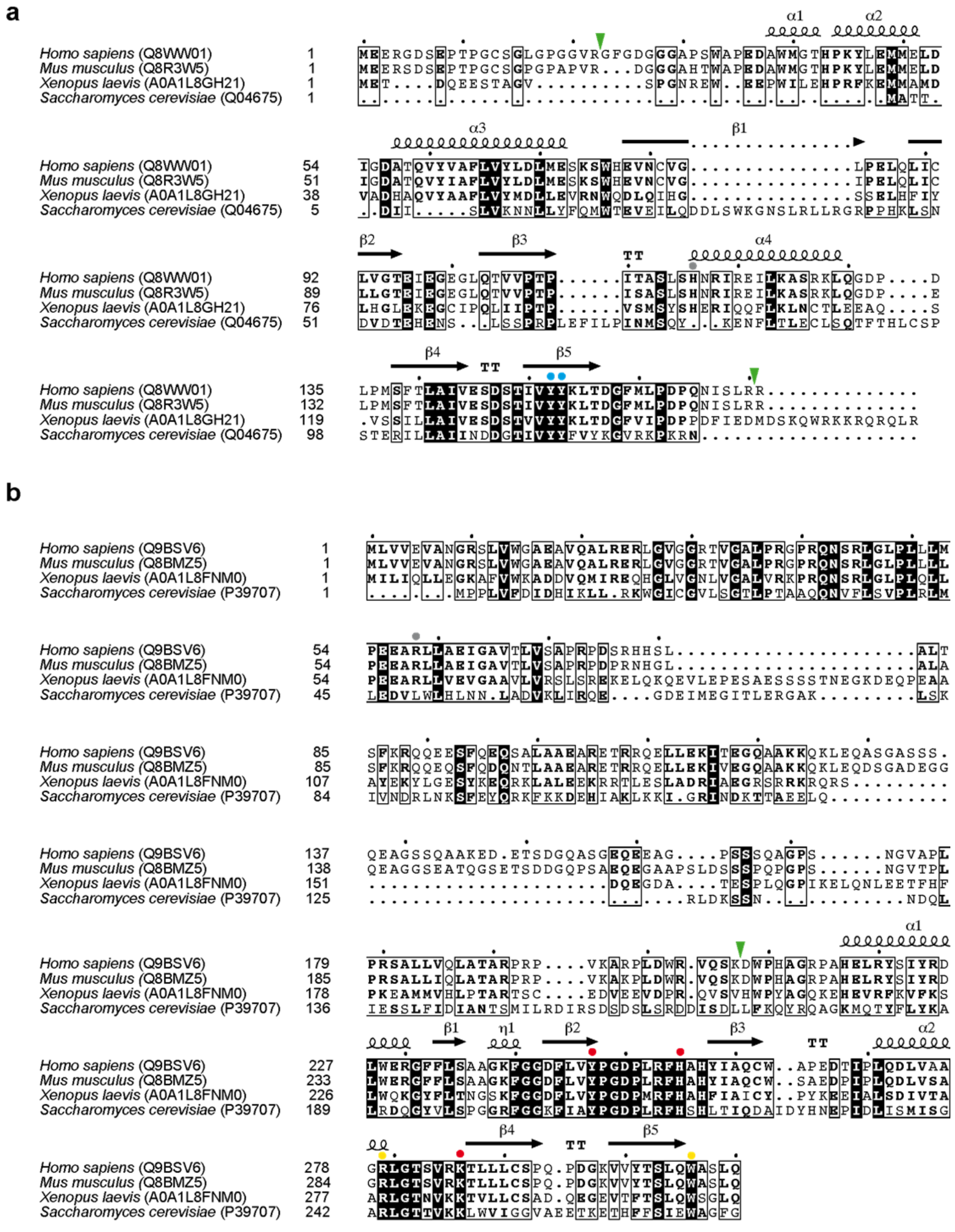

Supplementary Figure 5. **Sequence conservation of TSEN15 and TSEN34.** Sequence alignments were performed using Clustal Omega and colored by conservation using ESPript 3.0. **a** The TSEN15 sequence alignment includes orthologues from *Homo sapiens* (UniProtKB Q8WW01), *Mus musculus* (UniProtKB Q8R3W5), *Xenopus laevis* (UniProtKB A0A1L8GH21), and *Saccharomyces cerevisiae* (UniProtKB Q04675). **b** The TSEN34 sequence alignment includes orthologues from *Homo sapiens*

(UniProtKB Q9BSV6), *Mus musculus* (UniProtKB Q8BMZ5), *Xenopus laevis* (UniProtKB A0A1L8FNM0), and *Saccharomyces cerevisiae* (UniProtKB P39707). Tryptic sites identified from limited proteolysis experiments are shown by green arrow heads. The YY-motif is indicated by blue circles, residues of the catalytic triad are highlighted by red circles, and residues possibly involved in the cation-π-interaction are shown as yellow circles. Residues mutated in PCH (TSEN15^{H116Y}, TSEN34^{R58W}) are indicated by grey circles. Helices and strands are numbered sequentially according to the TSEN15–34 X-ray crystal structure and are indicated above the alignments. TT – β-turn.

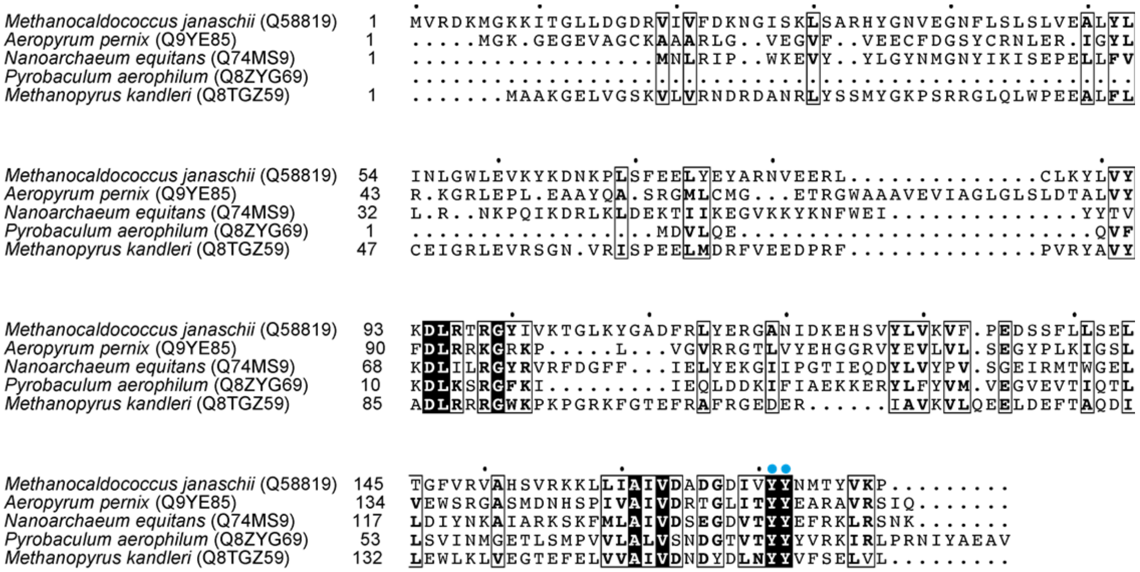

Supplementary Figure 6. **Sequence conservation of Archaeal α_4 and $(\alpha\beta)_2$ endonucleases highlighting the YY-motif.** Sequence alignments were performed using Clustal Omega and colored by conservation using ESPript 3.0. The sequence alignment includes orthologues from *Methanocaldococcus jannaschii* (UniProtKB Q58819), *Aeropyrum pernix* (UniProtKB Q9YE85), *Nanoarchaeum equitans* (UniProtKB Q74MS9), *Pyrobaculum aerophilum* (UniProtKB Q8ZYG6), and *Methanopyrus kandleri* (UniProtKB Q8TGZ5). The YY-motif is indicated by blue dots.

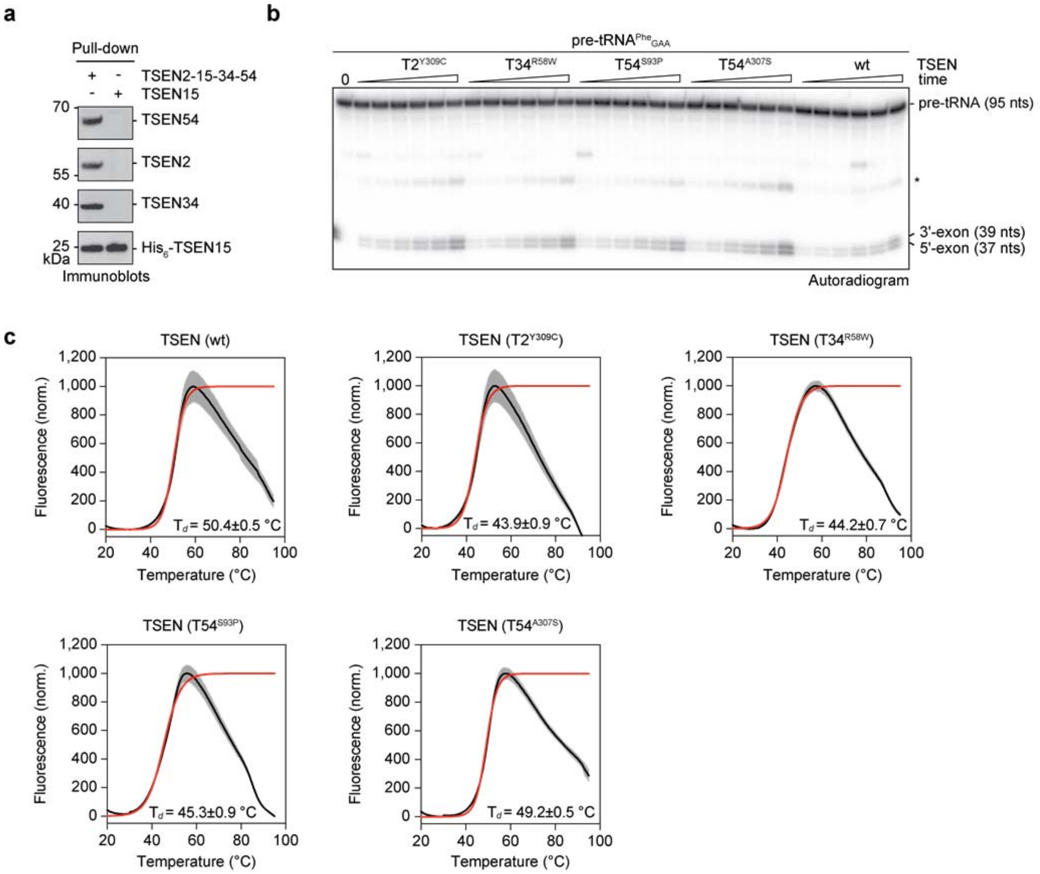

Supplementary Figure 7. **PCH mutations affect thermal stability but not activity of recombinant TSEN in vitro. a** Pull-down assay from HEK293T cells overexpressing TSEN subunits TSEN2, His_6-TSEN15, TSEN34, and TSEN54, or His_6-TSEN15 alone. Co-precipitated proteins were visualized by immunoblotting. **b** Pre-tRNA cleavage assay (time course) of radioactively labelled *S.c.* pre-$tRNA^{Phe}_{GAA}$ with wt or mutant TSEN complexes revealed by phosphorimaging. The asterisk indicates an intermediate cleavage product. **c** Representative thermal denaturation curves as shown in Fig. 4d of recombinant wt and mutant TSEN complexes derived from DSF with sigmoidal Boltzmann fits as red lines (black lines represent mean values). Grey zones show SDs from technical triplicates. Denaturation temperature (T_d) is presented with error of fit. Source data for **a** and **b** are provided as Source Data file.

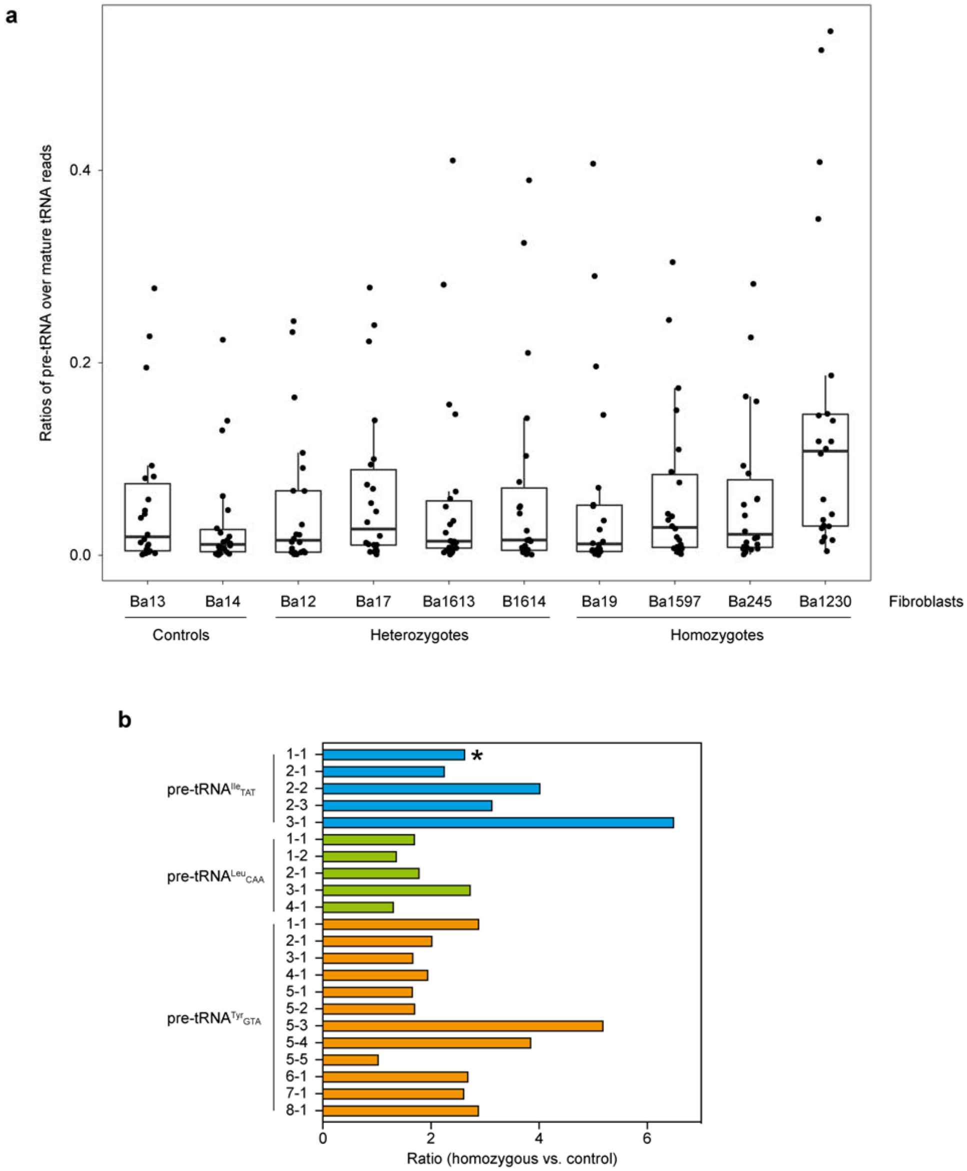

Supplementary Figure 8. **Hydro-tRNAseq reveals accumulation of intron-containing pre-tRNAs in *TSEN54* c.919G>T fibroblasts. a** Boxplots showing ratios of pre-tRNA over mature tRNA reads for all intron-containing tRNAs from hydro-tRNAseq of PCH patient-derived and control fibroblasts. Pre-tRNA reads for all samples and tRNAs are less abundant than mature tRNA reads (ratios < 1). The lowest median corresponds to a homozygous control, and the highest median to a homozygous TSEN54^{A307S} patient. Number of biologically independent samples: controls, n=2; heterozygotes, n=4; homozygotes, n=4. The dark horizontal line within the box denotes the median. The middle 50% of the data lie within the box. The lower and upper hinges correspond to the first and third quartiles (25th and 75th percentile, respectively). The upper whisker extends from the third quartile to the largest value no

further than 1.5 times the interquartile range (IQR; i.e., the distance between the first and third quartile). The lower whisker extends from the first quartile to the smallest value at most 1.5 times IQR from the hinge. Outliers are values beyond the whiskers and are plotted individually. **b** The ratio of hydro-tRNAseq reads mapped to pre-tRNAs over mature tRNAs was calculated for every intron-containing tRNA. The average ratio of all patients over the average ratio of all homozygous controls is shown. TSEN54 c.919G>T fibroblasts exhibit an increase of pre-tRNA/mature tRNA ratios for all intron-containing tRNAs. $tRNA^{Ile}_{TAT}$ isodecoders targeted by a 5' exon probe shown in Fig. 5b are highlighted in blue. $tRNA^{Ile}_{TAT}$1-1 targeted by an intron probe in Fig. 5b,d is marked with an asterisk. Source data for **a** and **b** are provided as Source Data file.

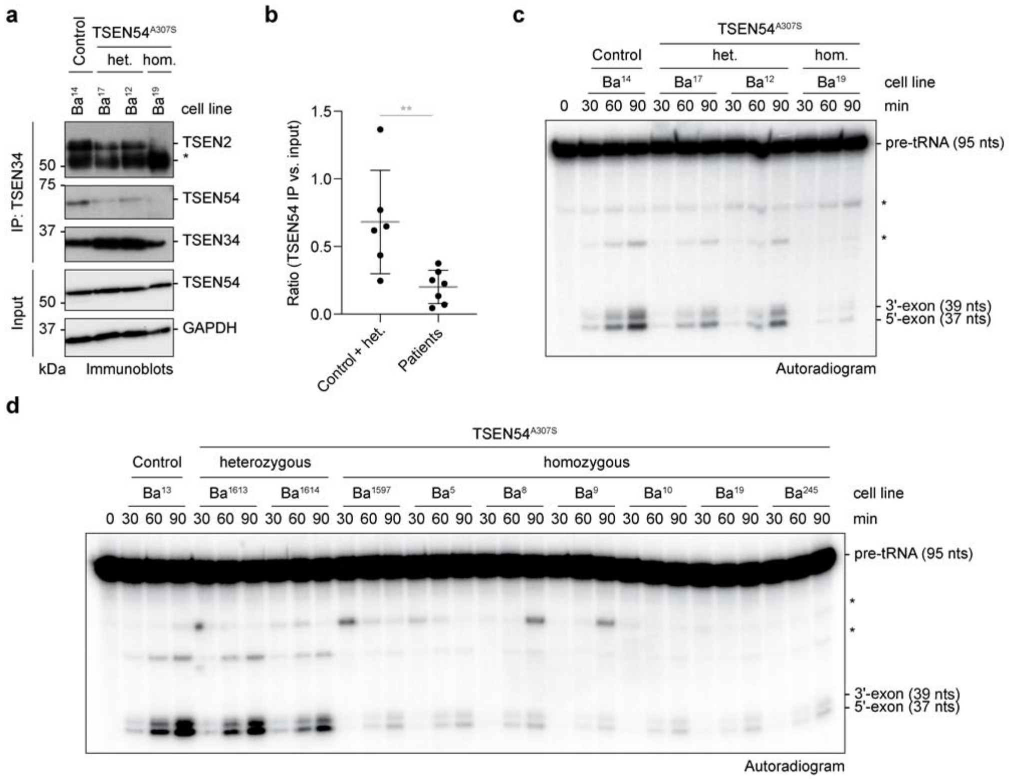

Supplementary Figure 9. **Reduced pre-tRNA cleavage activity in PCH patient-derived cell extracts is associated with altered composition of TSEN. a** Co-immunoprecipitation (IP) assay using an α-TSEN34 antibody with cell lysates derived from a fibroblast control cell line (Ba^{14}) and fibroblasts derived from a PCH patient (Ba^{19}) and the parents (Ba^{17} and Ba^{12}) analyzed by immunoblotting. The asterisk indicates the heavy chain of the α-TSEN34 antibody. GAPDH served as a loading control. **b** Statistical analysis of Fig. 6a and Supplementary Fig. 9a showing the ratio of immunoprecipitated (IP) TSEN54 versus input for control and heterozygous fibroblasts (n=6, different cell lines) and homozygous PCH patient fibroblasts (n=7, different cell lines). Unpaired Student´s *t*-test (two-tailed) revealed a significant difference in levels of co-immunoprecipitated TSEN54 in α-TSEN34 immunoprecipitates between the two groups (***P*=0.0092). Data are presented as mean values ±SD. **c** On-bead pre-tRNA cleavage assay (time course) with radioactively labelled *S.c.* $pre\text{-}tRNA^{Phe}_{GAA}$ and immunoprecipitated TSEN complexes (α-TSEN34 antibody-coupled resin) shown in (**a**). Unspecific bands are indicated by asterisks. **d** On-bead pre-tRNA cleavage assay (time course) with radioactively labelled *S.c.* $pre\text{-}tRNA^{Phe}_{GAA}$ and immunoprecipitated TSEN complexes (α-TSEN2 antibody-coupled resin) derived from control fibroblasts and from fibroblasts carrying heterozygous or homozygous

TSEN54 c.919G>T mutation. Unspecific bands are indicated by asterisks. Data are representative of at least two independent experiments. Source data for **a**, **c**, and **d**, are provided as Source Data file.

Supplementary Tables

Supplementary Table 1. **Masses of protein subunits and complexes observed in native MS spectra.** The experimentally determined and theoretically calculated masses as well as the mass differences are given. A larger mass difference (*) originates from incomplete desolvation and can be in part attributed to the high phosphorylation state of the TSEN54 subunit (Supplementary Fig. 1c).

Composition	Experimental mass (Da)	Theoretical mass (Da)	Δ mass (Da)
TSEN			
TSEN2–15–34–54	165573 ±130	164416	1157 (*)
unassigned	104865 ±48		
HSP70	71461 ±6	71432	29
TSEN15–34	52389 ±15	52350	39
TSEN15	18693 ±4	18698	-5
TSEN/CLP1			
unassigned	466075 ±127		
unassigned	417988 ±36		
TSEN2–15–34–54–2xCLP1	261096 ±182	259822	1274 (*)
TSEN2–15–34–54–1xCLP1	212967 ±98	212119	848 (*)
unassigned	123948 ±28		
unassigned	104818 ±6		
HSP70	71521 ±3	71432	89
TSEN15–34	52412 ±7	52350	62
CLP1	47776 ±5	47703	73
TSEN15	18704 ±0	18698	6

Subunits	Uniprot KB	Theoretical mass (Da)
TSEN15	Q8WW01	18698
TSEN34	Q9BSV6	33652
TSEN2	Q8NCE0	53247
TSEN54	Q7Z6J9	58819
CLP1	Q92989	47703
HSP70	Q9U639	71432

Supplementary Table 2. **Protein identification by LC-MS/MS.** The protein masses, the number of identified peptide sequences, the number of observed spectra, and the sequence coverage are given for TSEN subunits and CLP1 of purified TSEN, TSEN/CLP1 and proteolyzed TSEN15–34 complexes.

TSEN

Protein	UniProtKB	Mass (Da)	Peptide sequences (#)	Spectra (#)	Sequence coverage (%)
TSEN15	Q8WW01	18629	9	140	47.4
TSEN34	Q9BSV6	33631	53	907	100.0
TSEN2	Q8NCE0	53213	99	1228	98.5
TSEN54	Q7Z6J9	58783	67	771	84.8

TSEN/CLP1

Protein	UniProtKB	Mass (Da)	Peptide sequences (#)	Spectra (#)	Sequence coverage (%)
TSEN15	Q8WW01	18629	11	180	73
TSEN34	Q9BSV6	33631	58	1583	100
TSEN2	Q8NCE0	53213	86	1946	99
TSEN54	Q7Z6J9	58783	68	1132	95
CLP1	Q92989	47615	75	1314	100

Proteolyzed TSEN15–34

Protein	UniProtKB	Mass (Da)	Peptide sequences (#)	Spectra (#)	Sequence coverage (%)
TSEN15	Q8WW01	18629	9	92	87
TSEN34	Q9BSV6	33631	14	275	34

Supplementary Table 3. **Masses of proteolytic fragments of TSEN15 and TSEN34 obtained from denaturing MS.** The experimentally determined and theoretically calculated masses as well as the mass difference are given.

Protein fragment	Experimental mass (Da)	Theoretical mass (Da)	Δ mass (Da)
TSEN15 (residues 23 to 170)	16313.9±1.0	16314.7	-0.8
TSEN15 (residues 23 to 171)	16469.8±0.9	16470.9	-1.1
TSEN34 (residues 208 to 310)	11614.8±0.7	11615.2	-0.4

Supplementary Table 4. **Identification of proteolytic fragments of TSEN15 and TSEN34 by LC-MS/MS.** The position in the protein sequence, the sequence of observed tryptic peptides and the number of spectra for each peptide are given. The preceding and following amino acids are given for each peptide sequence.

TSEN15

Residues	Peptide sequence	# spectra
23-45	R.GFGDGGGAPSWAPEDAWMGTHPK.Y	22
46-74	K.YLEMMELDIGDATQVYVAFLVYLDLMESK.S + Oxidation (M)	4
75-118	K.SWHEVNCVGLPELQLICLVGTEIEGEGLQTVVPTPITASLSHNR.I	6
119-127	R.IREILKASR.K	7
121-127	R.EILKASR.K	7
128-154	R.KLQGDPDLPMSFTLAIVESDSTIVYYK.L	13
155-170	K.LTDGFMLPDPQNISLR.R	17
155-171	K.LTDGFMLPDPQNISLRR.-	16

TSEN34

Residues	Peptide sequence	# spectra
204-220	R.VQSKDWPHAGRPAHELR.Y	1
208-220	K.DWPHAGRPAHELR.Y	37
221-230	R.YSIYRDLWER.G	61
231-253	R.GFFLSAAGKFGGDFLVYPGDPLR.F	1
240-253	K.FGGDFLVYPGDPLR.F	6
254-279	R.FHAHYIAQCWAPEDTIPLQDLVAAGR.L	7
280-286	R.LGTSVRK.T	4
280-298	R.LGTSVRKTLLLCSPQPDGK.V	8
286-298	R.KTLLLCSPQPDGK.V	75
286-310	R.KTLLLCSPQPDGKVVYTSLQWASLQ.-	1
287-298	K.TLLLCSPQPDGK.V	68
287-310	K.TLLLCSPQPDGKVVYTSLQWASLQ.-	3
299-310	K.VVYTSLQWASLQ.-	3

Supplementary Table 5. **X-ray data collection, refinement, and validation statistics.** The structure of TSEN15–34 was determined from one protein crystal. Values in parentheses are given for highest-resolution shell.

	TSEN15–34 (PDB 6Z9U)
Data collection	
Space group	P 1 21 1
Cell dimensions	
a, *b*, *c* (Å)	34.85, 69.28, 94.79
α, β, γ (°)	90, 98.31, 90
Resolution (Å)	28.3 - 2.1(2.175 - 2.1)
R_{merge}	0.07428 (0.8863)
$I / \sigma I$	13.37 (1.44)
Completeness (%)	0.99 (0.99)
Redundancy	5.9 (5.9)
Refinement	
Resolution (Å)	28.3 - 2.1
No. reflections	25898 (2576)
R_{work} / R_{free}	19.18 (30.49) / 25.28 (36.27)
No. atoms	3636
Protein	3516
Ligand/ion	12
Water	120
B-factor (average, Å^2)	60.17
Protein	60.10
Ligand/ion	78.56
Water	60.32
R.m.s. deviations	
Bond lengths (Å)	0.009
Bond angles (°)	0.99
Validation	
Ramachandran plot	
Favored (%)	97
Allowed (%)	2.5
Outliers (%)	0.2
Rotamer outliers (%)	1
Clash score	9.26

Supplementary Table 6. **DSF data analyzed by ProteoPlex.** T_d – denaturing temperature.

TSEN complex	T_d - Boltzman (°C)	T_d - ProteoPlex (°C)	R^2 (fit to data)	R^2 (fit to 2-state unfolding)
TSEN (wt)	51.0	52.1	0.99973	0.99899
TSEN (T2^{Y309C})	44.8	46.3	0.99979	0.99955
TSEN (T34^{R58W})	44.1	46.5	0.99955	0.99902
TSEN (T54^{S93P})	46.5	48.7	0.99935	0.99818
TSEN ($\text{T54}^{\text{A307S}}$)	49.6	50.7	0.99978	0.99951

Supplementary Table 7. **List of patient-derived primary fibroblast cells used in this study.**

Cell line	Mutations	Description	Zygosity
Ba1	*TSEN54 c.919G / 919G*	control	homozygous
Ba2	*TSEN54 c.919G / 919G*	control	homozygous
Ba3	*TSEN54 c.919G / 919G*	control	homozygous
Ba5	*TSEN54 c.919G>T / 919G>T*	PCH2 patient	homozygous
Ba8	*TSEN54 c.919G>T / 919G>T*	PCH2 patient	homozygous
Ba9	*TSEN54 c.919G>T / 919G>T*	PCH2 patient	homozygous
Ba10	*TSEN54 c.919G>T / 919G>T*	PCH2 patient	homozygous
Ba12	*TSEN54 c.919G / 919G>T*	parent of Ba19	heterozygous
Ba13	*TSEN54 c.919G / 919G*	control	homozygous
Ba14	*TSEN54 c.919G / 919G*	control	homozygous
Ba15	*TSEN54 c.919G / 919G*	control	homozygous
Ba17	*TSEN54 c.919G / 919G>T*	parent of Ba19	heterozygous
Ba18	*TSEN54 c.919G>T / 919G>T*	PCH2 patient	homozygous
Ba19	*TSEN54 c.919G>T / 919G>T*	PCH2 patient	homozygous
Ba20	*TSEN54 c.919G>T / 923delC* p.(Pro318Gln fsX23)	PCH4 patient	compound heterozygous
Ba245	*TSEN54 c.919G>T / 919G>T*	PCH2 patient	homozygous
Ba1230	*TSEN54 c.919G>T / 919G>T*	PCH2 patient	homozygous
Ba1613	*TSEN54 c.919G / 919G>T*	parent of Ba1597	heterozygous
Ba1614	*TSEN54 c.919G / 919G>T*	parent of Ba1597	heterozygous
Ba1597	*TSEN54 c.919G>T / 919G>T*	PCH2 patient	homozygous
T1 (BAB3846)	*CLP1 c.419G / 419G>A*	parent of BAB3402	heterozygous
T3 (BAB3402)	*CLP1 c.419G>A / 419G>A*	patient	homozygous

Supplementary Table 8. **Primers used in this study.**

Primer	Sequence (5‘-3‘)
TSEN2_BamHI_for	TCTGTTTGGATCCATGGCAGAAGCAGTTTTCCATG
TSEN2_BamHI_His-TEV_for	GTTTGGATCCATGGGTCATCACCATCACCATCACGGTGAGAATCTTTATTTTCAG
TSEN2_His-TEV_for	CACGGTGAGAATCTTTATTTTCAGGGCATGGCAGAAGCAGTTTTCCATG
TSEN2_XbaI_rev	CAGGCTCTAGATTAAAGATCGTCTTGGTCACTCC
TSEN15_BamHI_His-TEV_for	GTTTGGATCCATGGGTCATCACCATCACCATCACGGTGAGAATCTTTATTTTCAG
TSEN15_His-TEV_for	CACGGTGAGAATCTTTATTTTCAGGGCATGGAGGAGCGCGGCGATTCC
TSEN15_NotI_rev	GAAAGCGGCCGCTCATCTTCTAAGAGAAATATTCTGAG
TSEN15_BamHI-His10_for	TCCGGGGATCCATGGGTCATCATCACCACCATCACCATCACCATCACGGTG
TSEN15_NotI_short_rev	AAAGCGGCCGCTCATCTTC
TSEN15_23-170_for	CTTTATTTTCAGGGCTTTGGCGACGGCGGTGGAG
TSEN15_23-170_rev	TTCGAAAGCGGCCGCTCATCTAAGAGAAATATTCTGAGGG
pMIDK_His_for	ATTTCTCTTAGATGAGCGGCCGCTTTCGAATCTAG
pMIDK_His_QS_rev	ACCGCCGTCGCCAAAGCCCTGAAAATAAAGATTCTCACC
TSEN34_AflII_for	AGCCACTTAAGATGCTGGTGGTGGAGGTGG
TSEN34_SphI_rev	GTACCGCATGCTCACTGCAGGCTGGCCCATTG
TSEN34_208-310_for	CTGTTTGGATCCATGGACTGGCCCCACGCCGGC
TSEN34_208-310_rev	GCAGGCTCTAGATTACTGCAGGCTGGCCCATTG
TSEN54_BamHI_for	TGTTTGGATCCATGGAGCCCGAGCCCGAGC
TSEN54_XbaI_rev	CAGGCTCTAGATCAGTGCCCCACATCCTGGG
CLP1_BamHI_for	TCTGTTTGGATCCATGGGAGAAGAGGCTAATGATG
CLP1_BamHI_His-TEV_for	GTTTGGATCCATGGGTCATCACCATCACCATCACGGTGAGAATCTTTATTTTCAG
CLP1_His-TEV_for	CACGGTGAGAATCTTTATTTTCAGGGCATGGGAGAAGAGGCTAATGATG
CLP1_XbaI_rev	CAGGCTCTAGACTACTTCAGATCCATGAACCGG
TSEN34_H255A_Quikchange_for	GCGATATAATGGGCGGCGAAGCGGAGGGGGTC
TSEN34_H255A_Quikchange_rev	GACCCCCTCCGCTTCGCCGCCCATTATATCGC
TSEN2_H377A_Quikchange_for	ATGACAGAATAACTTGCAGCGTAAAATGGAGGGCCTTTCCG
TSEN2_H377A_Quikchange_rev	CGGAAAGGCCCTCCATTTTACGCTGCAAGTTATTCTGTCAT
TSEN2_Y309C_Quikchange_for	GCCTTTTTCTTGGTCTGTGCTCTGGGATGTTTAAGTATTTAC
TSEN2_Y309C_Quikchange_rev	TAAACATCCCAGAGCACAGACCAAGAAAAAGGCCTCTTCTAG
TSEN54_A307S_Quikchange_for	GCGGCTGTCTGAAGACATGTTGGGGAAGG
TSEN54_A307S_Quikchange_rev	CCTTCCCCAACATGTCTTCAGACAGCCGC
TSEN54_S93P_Quikchange_for	TGCCCGCGGGAGGCTTCAACTCCACG
TSEN54_S93P_Quikchange_rev	CGTGGAGTTGAAGCCTCCCGCGGGCA
TSEN34_R58W_Quikchange_for	CGGCCAAGAGCCACGCCTCTTCGGG
TSEN34_R58W_Quikchange_rev	CCCGAAGAGGCGTGGCTCTTGGCCG
Pre-tRNA-PheGAA-BamHI_for	TCCGGGGATCCAATTAATACGACTCACTATAGCGGATTTAGCTCAGTTGGGAG
Pre-tRNA-PheGAA-HindIII_rev	ACGCCAAGCTTCCTGGTGCGAATTCTGTGGATCG
Pre-tRNA-PheGAA_CC_for	AGAAAAAACTTCGGTCAACTTATCTGGAGGTCCTGTG
Pre-tRNA-PheGAA_CC_rev	CACAGGACCTCCAGATAAGTTGACCGAAGTTTTTTCT
Pre-tRNA-PheGAA_GG/GC_for	AGTTGGGAGAGCGCCAGAGTGAAGAAAAAACTTC
Pre-tRNA-PheGAA_GG/GC_rev	GAAGTTTTTTCTTCACTCTGGCGCTCTCCCAACT
tRNA-PheGAA_Quikchange_for	GAGCGCCAGACTGAAGATCTGGAGGTCCTGTG
tRNA-PheGAA_Quikchange_rev	CACAGGACCTCCAGATCTTCAGTCTGGCGCTC
tRNA-PheGAA_Q5_for	ATCTGGAGGTCCTGTGTTC
tRNA-PheGAA_Q5_rev	CTTCAGTCTGGCGCTCTC
Pre-tRNA-TyrGTA-8-1_1°_for	GCTAGGGGTCCTTTCGATAG
Pre-tRNA-TyrGTA-8-1_1°_rev	TTCAAAACTTTCGTCCTTCGAG
Pre-tRNA-TyrGTA_8-1_2°_for	TCCGGGGATCCAATTAATACGACTCACTATAGGTTCGATAGCTCAGTTGGTAG
Pre-tRNA-TyrGTA_8-1_2°_rev	ACGCCAAGCTTCCTGGTGGTTCGAGCCGGATTCG
Pre-tRNA-TyrGTA_8-1_CC_for	GAGGACTGTAGGTTCATTAAACTAACGCATCCTTAGGTC
Pre-tRNA-TyrGTA_8-1_CC_rev	GACCTAAGGATGCGTTAGTTTAATGAACCTACAGTCCTC
Pre-tRNA-TyrGTA_8-1_GG/GC_for	CAGTTGGTAGAGCGGAGGAGTGTAGGTTCATTAAAC
Pre-tRNA-TyrGTA_8-1_GG/GC_rev	GTTTAATGAACCTACACTCCTCCGCTCTACCAACTG
CasI_For	AACGCTCTATGGTCTAAAGATTTAAATCGACCTACTCCGGAATATTAATAGATC
CasI_Rev	AAACGTGCAATAGTATCCAGTTTATTTAAATGGTTATGATAGTTATTGCTCAGC
CasII_For	AAACTGGATACTATTGCACGTTTAAATCGACCTACTCCGGAATATTAATAGATC

**Primer**	**Sequence (5‘-3‘)**
CasII_Rev	AAACATCAGGCATCATTAGGTTTATTTAAATGGTTATGATAGTTATTGCTCAGCG
CasIII_For	AAACCTAATGATGCCTGATGTTTAAATCGACCTACTCCGGAATATTAATAGATC
CasIII_Rev	AAACTAAGCTATGTGAACCGTTTATTTAAATGGTTATGATAGTTATTGCTCAGCG
CasIV_For	AAACGGTTCACATAGCTTAGTTTAAATCGACCTACTCCGGAATATTAATAGATC
CasOmega_Rev	AACCCCGATTGAGATATAGATTTATTTAAATGGTTATGATAGTTATTGCTCAGCG
TSEN2_H377A_Quikchange_For	GTCCGCCGTTCTACGCCGCGAGCTATAGCG
TSEN2_H377A_Quikchange_Rev	CGCTATAGCTCGCGGCGTAGAACGGCGGAC
TSEN34_H255A_Quikchange_For	CGATCCGCTGCGTTTCGCTGCGCACTATATCGC
TSEN34_H255A_Quikchange_Rev	GCGATATAGTGCGCAGCGAAACGCAGCGGATCG
