# Supplementary material for: Assembly defects of human tRNA splicing endonuclease contribute to impaired pre-tRNA processing in pontocerebellar hypoplasia
Source: Nat Commun. 2021 Sep 28;12:5610. doi: 10.1038/s41467-021-25870-3 (PMC8479045; doi:10.1038/s41467-021-25870-3)
Supplement: Supplementary file 3 — Reporting Summary [file 41467_2021_25870_MOESM3_ESM.pdf]

nature portfolio

Corresponding author(s): Javier Martinez, Simon Trowitzsch

Last updated by author(s): Aug 10, 2021

Reporting Summary

Nature Portfolio wishes to improve the reproducibility of the work that we publish. This form provides structure for consistency and transparency in reporting. For further information on Nature Portfolio policies, see our Editorial Policies and the Editorial Policy Checklist.

Statistics

For all statistical analyses, confirm that the following items are present in the figure legend, table legend, main text, or Methods section.

n/a	Confirmed	
☐	☒	The exact sample size (n) for each experimental group/condition, given as a discrete number and unit of measurement
☒	☐	A statement on whether measurements were taken from distinct samples or whether the same sample was measured repeatedly
☐	☒	The statistical test(s) used AND whether they are one- or two-sided<br>*Only common tests should be described solely by name; describe more complex techniques in the Methods section.*
☒	☐	A description of all covariates tested
☒	☐	A description of any assumptions or corrections, such as tests of normality and adjustment for multiple comparisons
☐	☒	A full description of the statistical parameters including central tendency (e.g. means) or other basic estimates (e.g. regression coefficient) AND variation (e.g. standard deviation) or associated estimates of uncertainty (e.g. confidence intervals)
☐	☒	For null hypothesis testing, the test statistic (e.g. F, t, r) with confidence intervals, effect sizes, degrees of freedom and P value noted<br>*Give P values as exact values whenever suitable.*
☒	☐	For Bayesian analysis, information on the choice of priors and Markov chain Monte Carlo settings
☒	☐	For hierarchical and complex designs, identification of the appropriate level for tests and full reporting of outcomes
☒	☐	Estimates of effect sizes (e.g. Cohen's d, Pearson's r), indicating how they were calculated

Our web collection on statistics for biologists contains articles on many of the points above.

Software and code

Policy information about availability of computer code

Data collection	X-ray crystallography: CBFlib v0.9.5 (Dectris, Crystallography Binary File (CBF) version 1.7.10). Mass spectrometry: XCalibur v4.2.47 (Thermo Scientific, vendor-specific software for Q Exactive Plus Hybrid Quadrupole), MassLynx v4.1 (Waters Corporation, vendor-specific software for Quadrupole Time-of-flight Ultima mass spectrometer modified for transmission of high mass complexes).
Data analysis	X-ray crystallography: XDS (built 20180126 and built 20180307), Coot (0.8.2 EL and 0.9.3 EL), Phenix (version 1.10.1-2155), MacPyMOL (v1.7.2.0). DSF and fluorescence anisotropy: Prism 5 and Prism 8 (Graphpad Software). SEC-MALS: Astra V 5.3.4.13 (ASTRA software package, Wyatt Technology Corporation). Mass spectrometry: Mascot search engine 2.5.1 (Matrix Science), MassLynx v4.1 (Waters Corporation), Massing (Morgner & Robinson, 2012, Anal Chem, 84, 2939-48). Hydro-tRNAseq: cutadapt (http://journal.embnet.org/index.php/embnetjournal/article/view/200/458), R (version 4.0.4, https://www.r-project.org/), Python (version 3.7, http://www.python.org), Perl (version 5.18.4, https://www.perl.org/); figures were produced using the R package ggplot2 and Prism 8 (Graphpad Software).

For manuscripts utilizing custom algorithms or software that are central to the research but not yet described in published literature, software must be made available to editors and reviewers. We strongly encourage code deposition in a community repository (e.g. GitHub). See the Nature Portfolio guidelines for submitting code & software for further information.

Data

Policy information about availability of data

All manuscripts must include a data availability statement. This statement should provide the following information, where applicable:

- Accession codes, unique identifiers, or web links for publicly available datasets
- A description of any restrictions on data availability
- For clinical datasets or third party data, please ensure that the statement adheres to our policy

Atomic coordinates and structure factors were deposited to the Protein Data Bank (http://www.rcsb.org) under accession number PDB ID 6Z9U (https://www.rcsb.org/structure/6Z9U). The mass spectrometry proteomics data were deposited to the ProteomeXchange Consortium via the PRIDE partner repository with the dataset identifier PXD019034 (https://www.ebi.ac.uk/pride/archive/projects/PXD019034). Hydro-tRNAseq data were deposited with the Gene Expression Omnibus (GEO) repository under accession code GSE151236 (https://www.ncbi.nlm.nih.gov/geo/query/acc.cgi?acc=GSE151236). Source data for Figs. 1-6 and Extended Data Figs. 1-6 are provided with this paper.

Field-specific reporting

Please select the one below that is the best fit for your research. If you are not sure, read the appropriate sections before making your selection.

☒ Life sciences ☐ Behavioural & social sciences ☐ Ecological, evolutionary & environmental sciences

For a reference copy of the document with all sections, see nature.com/documents/nr-reporting-summary-flat.pdf

Life sciences study design

All studies must disclose on these points even when the disclosure is negative.

Sample size	No sample size calculations were performed. Available samples of PCH patients were used for analytical purposes in a targeted biochemical analysis. Sample sizes were dependent on the availability of PCH patient and control material and are indicated at the relevant text passages and figure captions in the manuscript.
Data exclusions	No data were excluded from analyses.
Replication	All replications were successful. Number of repetitions are stated in each figure caption.
Randomization	No randomizations were taken. Covariates were not controlled, since only a limited number of PCH patients were available for targeted biochemical analyses. Patients' characteristics were not adjusted and no comparisons of treatments in randomized clinical trials were performed.
Blinding	Blinding was not relevant to any of the experiments presented in the manuscript, because only skin biopsies of individuals carrying a PCH mutation were chosen for targeted biochemical analyses. Only a limited number of PCH patients were available. No clinical trials with different cohorts of patients were performed.

Reporting for specific materials, systems and methods

We require information from authors about some types of materials, experimental systems and methods used in many studies. Here, indicate whether each material, system or method listed is relevant to your study. If you are not sure if a list item applies to your research, read the appropriate section before selecting a response.

Materials & experimental systems

n/a	Involved in the study	
☐	☒	Antibodies
☐	☒	Eukaryotic cell lines
☒	☐	Palaeontology and archaeology
☒	☐	Animals and other organisms
☐	☒	Human research participants
☒	☐	Clinical data
☒	☐	Dual use research of concern

Methods

n/a	Involved in the study	
☒	☐	ChIP-seq
☒	☐	Flow cytometry
☒	☐	MRI-based neuroimaging

Antibodies

Antibodies used	Antibodies used in western blotting (WB) and immunoprecipitation (IP) were: anti-TSEN15 (rabbit polyclonal, Atlas Antibodies, HPA029237; WB dilution 1:1000), anti-TSEN34 (IP, WB dilution 1:5000), anti-TSEN54 (WB dilution 1:5000), anti-TSEN2 (IP, WB dilution 1:5000), anti-GAPDH (rabbit monoclonal, 14C10, Cell Signaling Technology, #2118; WB dilution 1:1000), anti-beta-actin

	(mouse monoclonal, clone AC-74, Sigma-Aldrich, A2228; WB dilution 1:3000), anti-mouse IgG–peroxidase conjugate (secondary goat polyclonal, Sigma-Aldrich, A2554; WB dilution 1:20.000), anti-rabbit IgG–peroxidase conjugate (secondary goat polyclonal, Sigma-Aldrich, AP307P; WB dilution 1:20.000), anti-polyHistidine (mouse monoclonal, clone HIS-1, Sigma-Aldrich, H1029; IP).
Validation	Primary anti-TSEN15 antibody is validated for immunoblotting of overexpressed recombinant human TSEN15 in human cells (www.atlasantibodies.com), anti-polyHistidine antibody is validated for immunoblotting and immunoprecipitation of polyhistidine-tagged fusion proteins (www.sigmaaldrich.com), anti-beta-actin antibody is validated for immunoblotting of endogenous human beta-actin in human fibroblasts (www.sigmaaldrich.com), anti-GAPDH antibody is validated for immunoblotting of endogenous human GAPDH in various cell lines (https://www.cellsignal.de). Primary antibodies anti-TSEN34, anti-TSEN54, and anti-TSEN2, were purified by affinity purification via peptides used for raising the antibodies and validated for immunoblotting and immunoprecipitation of the respective human proteins (Gramsch Laboratories, Schwabhausen, Germany; Hanada et al., 2013, Nature, 495, 474–480; and this study).

Eukaryotic cell lines

Policy information about cell lines

Cell line source(s)	293 (DSMZ, ACC 305), 293T (DSMZ, ACC 635), Spodoptera frugiperda (Invitrogen, 11497013), human primary fibroblasts
Authentication	Cell lines were authenticated morphologically when cultured. Cells were kept in frozen vials from original stocks and not passaged more than 30 passages. Patient mutations in human primary fibroblasts were validated by sequencing.
Mycoplasma contamination	All human cell lines regularly tested negative for mycoplasma contamination. The Spodoptera frugiperda 21 cell line was not tested for mycoplasma contamination.
Commonly misidentified lines (See ICLAC register)	No misidentified cell lines were used.

Human research participants

Policy information about studies involving human research participants

Population characteristics	No covariate-relevant population characteristics of human research participants are stated (see Life sciences study design in this reporting summary). Primary fibroblast cell lines were generated from skin biopsies taken for diagnostic procedures. As soon as DNA diagnostics became available, patient DNA was subjected to genetic analyses. DNA sequencing confirmed the diagnosis and the PCH mutations were confirmed in the fibroblast cell lines.
Recruitment	Patients suspected for PCH were submitted to the pediatric neurology of the Academic Medical Centre (AMC) for diagnostics. Only skin biopsies of patients with the relevant PCH mutation, their healthy parents, or controls were considered in this study. Biochemical results were unbiased for the results of the assays.
Ethics oversight	All procedures were performed with full consent of the legal representative and approval of the Institutional Review Board (IRB), Amsterdam UMC, The Netherlands (#W17_090# 17.098).

Note that full information on the approval of the study protocol must also be provided in the manuscript.
